# Serine Metabolism Regulates the Replicative Senescence of Human Dental Pulp Cells through Histone Methylation

**DOI:** 10.3390/cimb46040179

**Published:** 2024-03-24

**Authors:** Shuhan Zhou, Jingyao Cui, Yu Shi

**Affiliations:** 1 State Key Laboratory of Oral Diseases & National Center for Stomatology & National Clinical Research Center for Oral Diseases, West China Hospital of Stomatology, Sichuan University, Chengdu 610041, China; zhoushuhan@stu.scu.edu.cn (S.Z.); cuijingyao2021@stu.scu.edu.cn (J.C.); 2 Department of Endodontics, West China School of Stomatology, Sichuan University, Chengdu 610041, China

**Keywords:** PHGDH, replicative senescence, human dental pulp cell, serine, methylation

## Abstract

Tissue regeneration therapy based on human dental pulp cells (hDPCs) faces the distinct challenge of cellular senescence during massive expansion in vitro. To further explore the regulatory mechanism of cellular senescence in hDPCs, we conduct experiments on young cells (Passage 5, P5) and replicative senescent (Passage 12, P12) hDPCs. The results confirm that hDPCs undergo replicative senescence with passaging, during which their ability to proliferate and osteogenic differentiation decreases. Notably, during replicative senescence, phosphoglycerate dehydrogenase (PHGDH), the key enzyme of the serine synthesis pathway (SSP), is significantly downregulated, as well as S-adenosylmethionine (SAM) levels, resulting in reduced H3K36me3 modification on Sirtuin 1 (*SIRT1*)and Runt-related transcription factor 2 (*RUNX2*) promoters. Inhibition of PHGDH leads to the same phenotype as replicative senescence. Serine supplementation fails to rescue the senescence phenotype caused by replicative senescence and inhibitors, in which folate metabolism-related genes, including serine hydroxymethyl transferase 2 (*SHMT2*), methylenetetrahydrofolate dehydrogenase 1(*MTHFD1*), methylenetetrahydrofolate dehydrogenase 2(*MTHFD2*), are notably decreased. Our research raised a possibility that PHGDH may be involved in cellular senescence by affecting folate metabolism and histone methylation in addition to serine biosynthesis, providing potential targets to prevent senescence.

## 1. Introduction

Human dental pulp cells (hDPCs) are used in tissue engineering and regenerative medicine due to their multilineage differentiation potential, abundant sources, and ease of extraction [1,2,3]. However, tissue regeneration therapy based on hDPCs requires extensive expansion in the clinical application. Cultured primary cells do not grow infinitely, but undergo only a limited number of cell divisions, in a process called cellular senescence [4]. The aging of mesenchymal stem cells affects their differentiation potential, ability to regulate the immune system, and thus their therapeutic potential [5]. Therefore, investigating the regulatory mechanism of cellular senescence in hDPCs may provide potential intervention methods to improve the efficacy of tissue regeneration therapy.

Metabolic changes are closely linked to aging and disease. For example, high blood sugar can cause aging of the eyes and kidneys, eventually leading to diabetic complications [6]. Metabolic intervention can extend lifespan. Restriction of methionine and tryptophan has been shown to extend lifespan in animal models [7,8]. In human fibroblasts, methionine restriction also delays cellular senescence and extends replicative lifespan [9]. Epigenetic changes, such as chromatin methylation and acetylation, are one of the important mechanisms by which metabolism affects aging [7].

Serine essential for cell proliferation supports the synthesis of proteins, amino acids, and glutathione. It also contributes to the synthesis of nucleotides and nicotinamide adenine dinucleotide phosphate (NADPH) as a one-carbon donor for one-carbon metabolism [10]. Serine can be obtained from exogenous sources or de novo serine synthesis involving three enzymes: phosphoglycerate dehydrogenase (PHGDH), phosphoserine aminotransferase 1 (PSAT1), and phosphoserine phosphatase (PSPH). Through one-carbon metabolism, serine also promotes S-adenosylmethionine (SAM) synthesis, which is a donor for methylation reactions [11]. It has been reported that serine metabolism affects age-related senescence of hDPCs by regulating *P16* DNA methylation [12]. Another study has shown that serine and PHGDH, a key enzyme in serine metabolism, affect the senescence of vascular endothelial cells by regulating the stability of pyruvate kinase M2 (PKM2) [13].

However, it remains unclear whether serine metabolism affects hDPCs senescence by regulating histone methylation. In this study, we investigated for the first time the relationship between serine metabolism and replicative senescence of hDPCs. We demonstrated that hDPCs undergo replicative senescence during passage, and their ability to proliferate and differentiate decreases. During cellular senescence serine metabolism and SAM were decreased, thereby regulating the expression of Sirtuin 1 (*SIRT1*) and Runt-related transcription factor 2 (*RUNX2*) through H3K36me3. PHGDH inhibition phenocopied the replicative senescence, both of which involved decreased folate metabolism and could not be rescued by serine supplementation. Our study advances the understanding of replicative senescence and provides a reference for delaying replicative senescence of hDPCs when applied in tissue regeneration therapy.

## 2. Materials and Methods

### 2.1. Cell Isolation and Culture

All subjects gave their informed consent for inclusion before they participated in the study. The study was conducted following the Declaration of Helsinki, and the protocol was approved by the Ethics Committee of West China Hospital of Stomatology (WCHSIRB-CT-2023-412). Human dental pulp tissue was collected from extracted healthy wisdom teeth or orthodontic teeth from patients aged 18–26 years old. Four subjects were included: a 22-year-old male, a 23-year-old female, a 24-year-old female, and a 26-year-old female. Teeth were placed in pre-cooled PBS containing 1% P/S immediately after extraction. They were rinsed 3 times with PBS containing 1% P/S to clean the surface. Sterile cotton balls and sterile drapes were used to completely wrap the tooth. A bone mallet was used to split the tooth. As much of the pulp tissue as possible was removed and minced into small pieces. The pulp tissue was digested with 0.1% type I collagenase (BioFroxx, Einhausen, Germany) for 1 h in a 37 °C incubator, removed and shaken every 10 min. The cell pellet obtained was resuspended and grown in MEM Alpha (Thermo Fisher Scientific, Waltham, MA, USA) containing 10% FBS, and 1% penicillin/streptomycin solution. The medium was changed on the fourth day. Young (Passage 5, P5) and senescent (Passage 12, P12) hDPCs were used for subsequent experiments. In the serine/glycine starvation experiment, the control medium used DMEM (Thermo Fisher Scientific, Waltham, MA, USA), and the serine/glycine-free medium was MEM (#11090, Thermo Fisher Scientific, Waltham, MA, USA) supplemented with 10% FBS, 2 mM glutamine, D-glucose (to a final concentration of 25 mM), vitamins (#11120, Thermo Fisher Scientific, Waltham, MA, USA) and 1% penicillin/streptomycin.

### 2.2. Western Blot

Cells were lysed by RIPA lysis buffer (Beyotime, Shanghai, China) on ice. Then it was mixed with SDS sample buffer and boiled for 5 min. Supernatants were separated by 7.5–15% PAGE Gel Fast Preparation Kit (EpiZyme, Shanghai, China) and transferred to 0.22 μM polyvinylidene fluoride (PVDF) membrane (Millipore, Darmstadt, Germany). The membrane was blocked with 5% BSA and incubated with primary antibody at 4 °C overnight. The membrane was incubated with horseradish peroxidase (HRP)-conjugated IgG secondary antibody for 1 h and then detected using an ECL Chemiluminescence Detection Kit (Vazyme, Nanjing, China).

### 2.3. Antibody

All antibodies used in the experiments were purchased. Actin (AF5003, 1:1000) and LMNB1 (AF1408, 1:1000) were purchased from Beyotime; P21 (10355-1-AP, 1:1000) was purchased from Proteintech (Wuhan, China); PHGDH (66350, 1:1000), yH2AX (9718, 1:800) and Ki67 (9129, 1:400) were purchased from Cell Signaling Technology (Danvers, MA, USA); PSAT1 (53804, 1:1000) was purchased from Signalway Antibody (Greenbelt, MD, USA); PSPH (DF12711, 1:1000) was purchased from Affinity Biosciences (Cincinnati, OH, USA); H3 (ab1791, 1:1000), H3K4me3 (ab213224, 1:1000), H3K9me3 (ab176916, 1:1000), H3K27me3 (ab192985, 1:1000), H3K36me3 (ab282572, 1:1000) were purchased from Abcam (Waltham, MA, USA).

### 2.4. RNA Extraction and Quantitative Reverse Transcription PCR (RT-qPCR)

RNA was isolated using TRIzol (Invitrogen, Waltham, MA, USA) and its absorbance was measured with a NanoDrop 2000 spectrophotometer (Thermo Fisher Scientific, Waltham, MA, USA). Reverse Transcriptase Kit (Vazyme Nanjing, China) was used to synthesize cDNA. cDNA was quantified using SYBR qPCR Master Mix system (Q712-02, Vazyme, Nanjing, China) under the following conditions: 95 °C for 30 s and then 39 cycles of 95 °C for 10 s and 60 °C for 30 s, followed by a dissociation program at 95 °C for 15 s, 60 °C for 60 s, and 95 °C for 15 s. β-actin was used as an internal reference. The 2^−ΔΔCt^ method was used to quantify the levels of gene expression. Primers are listed in Table 1.

### 2.5. S-Adenosylmethionine (SAM) Assay

The concentration of SAM was detected using an ELISA Kit (CEG414Ge, Cloud-Clone Corp, Wuhan, China) following the instructions.

### 2.6. Cell Counting Kit-8 (CCK-8) Assay

5000 cells per well were seeded into 96-well plates. Cells were treated with PHGDH inhibitors, NCT-503 and CBR-5884, at different concentrations for 48 h. After 2-day treatment, 10 μL of cck8 (k1018, APExBIO, Houston, TX, USA) solution was added per well, then incubated for 1.5 h. A microplate reader (Multiskan Sky, Thermo Fisher Scientific) was used to read the absorbance at 450 nm.

### 2.7. 5-Ethynyl-2′-Deoxyuridine (EdU) Incorporation Assay

Cells were seeded into confocal dishes. Click-iT EdU Alexa Fluor 488 Imaging Kit (Thermo Fisher Scientific) was used following the instructions. A confocal microscope (Olympus SpinSR, Tokyo, Japan) was used for image acquisition. Positive cells were quantified by ImageJ.

### 2.8. Colony Formation Assay

1000 cells per well were seeded into 6-well plates. Cells were treated with PHGDH inhibitors (NCT-503 and CBR-5884) for 48 h or supplemented with serine at different concentrations (0.3 mM, 1 mM) during passage. 0.1% crystal violet was used for staining.

### 2.9. Osteogenic Induction

Cells were seeded into 6-well plates and cultured for 3 days in a complete medium supplemented with 10 nM dexamethasone, 50 mg/mL ascorbic acid 2-phosphate, and 10 mM β-glycerophosphate (all from Sigma-Aldrich, Louis, MO, USA). BCIP/NBT Alkaline Phosphatase Color Development Kit (Beyotime) was used for staining.

### 2.10. Senescence-Associated β-Galactosidase (SA-β-Gal) Staining

Cells were seeded into 24-well plates. Cells were treated with PHGDH inhibitors (NCT-503 and CBR-5884) for 48 h or supplemented with serine at different concentrations (0.3 mM, 1 mM) during passage or treated with both PHGDH inhibitors and serine for 48 h in serine/glycine-free medium. Senescence β-Galactosidase Staining Kit (Beyotime) was used for staining.

### 2.11. Immunofluorescence

Cells were seeded into confocal dishes. Cells were fixed with 4% paraformaldehyde, permeabilized with 0.25% triton, and blocked with 5% BSA. Cells were incubated with primary antibody at 4 °C overnight and then incubated with Alexa Fluor-conjugated secondary antibody (Invitrogen) in the dark for 1 h. Cells were incubated with DAPI for 5 min. A confocal microscope (Olympus SpinSR) was used for image acquisition. Positive cells were quantified by ImageJ.

### 2.12. Chromatin Immunoprecipitation (ChIP)

ChIP was conducted using the ChIP Assay Kit (P2078, Beyotime). Briefly, cross-linking was performed with 1% formaldehyde and terminated by 0.125 M glycine. Cells were collected and lysed. DNA was sheared by sonication and incubated with primary antibodies at 4 °C overnight. The complex was precipitated by Protein A + G agarose/salmon spermatozoa DNA. The sample was washed and heated at 65 °C for 4 h to reverse crosslink. OMEGA E.Z.N.A. Cycle Pure Kit was used to purify DNA. The primers used in subsequent qPCR are listed in Table 1.

### 2.13. Statistical Analysis

GraphPad Prism9 (v.9.0.0) was used for statistical analysis. Data was reported as means ± SD. Each experiment was repeated three times independently. The Shapiro-Wilk test was used first to test for normality. For two groups of data, if normality and homogeneity of variances are satisfied, an unpaired *t*-test is used. If normality is not satisfied or variances are not equal, the Mann-Whitney U test is used. For three or more groups of data that all satisfy normality and homogeneity of variances, one-way/two-way ANOVA is used, and multiple comparisons are performed using the Turkey test. Differences were statistically significant when the *p* value < 0.05.

## 3. Results

### 3.1. Human Dental Pulp Cells Undergo Senescence during Passage

**Figure 1:**
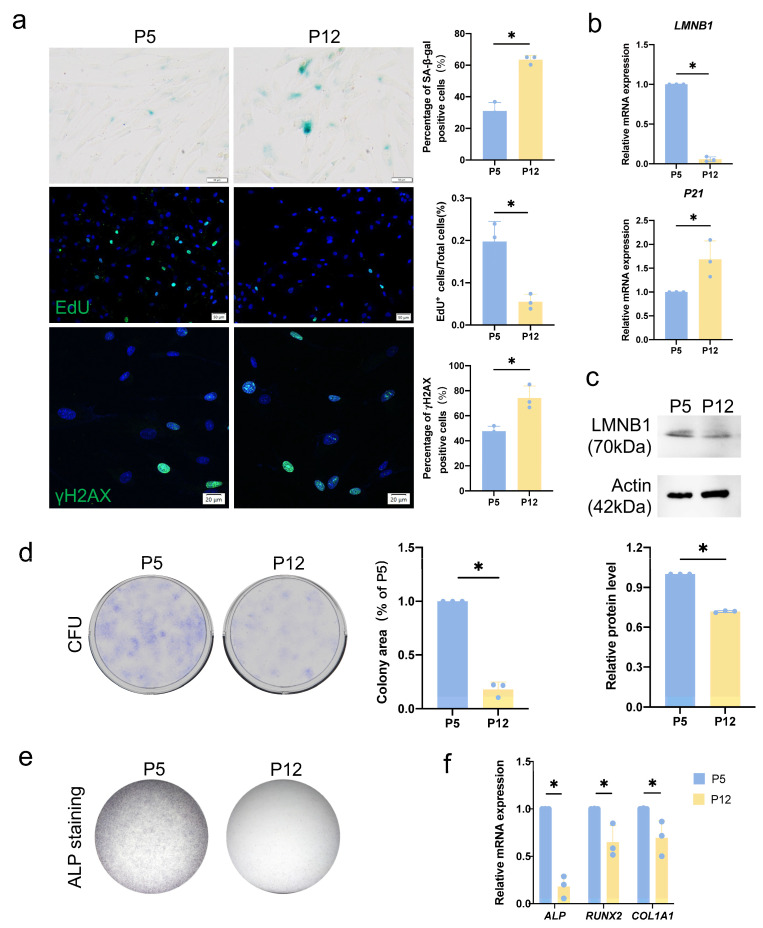
Human dental pulp cells (hDPCs) undergo senescence during passage. (**a**) Effect of passage (Passage 5 to Passage 12) on human dental pulp cells (hDPCs) senescence was determined by senescence-associated β-galactosidase (SA-β-gal) staining, 5-Ethynyl-2′-deoxyuridine (EdU) staining, and phosphorylated H2AX (yH2AX) staining. In SA-β-gal staining, blue-stained cells are positive cells. In EdU staining and yH2AX staining, the blue color in the nucleus shows DAPI staining, the green color in the nucleus shows EdU or yH2AX staining. (**b**) Analysis of Lamin B1 (*LMNB1*) and *P21* transcripts in different passages (P5 and P12) of hDPCs by quantitative reverse transcription PCR (RT-qPCR). (**c**) Analysis of LMNB1 expression in different passages (P5 and P12) of hDPCs by western blot. (**d**) Crystal violet staining determined the colony formation in different passages (P5 and P12) of hDPCs at 10 days. (**e**) alkaline phosphatase (ALP) staining after osteogenic induction for 3 days in different passages (P5 and P12) of hDPCs. (**f**) RT-qPCR analysis of *ALP*, *RUNX2*, and *COL1A1* transcripts after osteogenic induction for 3 days in different passages (P5 and P12) of hDPCs. Data were expressed as means ± SD, *n* = 3 independent experiments. * *p* < 0.05.

To confirm the changes in human dental pulp cells (hDPCs) during passage, we first detected the senescent phenotype. We observed an increased percentage of senescent cells in Passage 12 (P12) hDPCs by increased senescence-associated β-galactosidase (SA-β-gal) staining, phosphorylated H2AX (yH2AX) staining, and decreased 5-Ethynyl-2′-deoxyuridine (EdU) staining (Figure 1a). In addition, the increased expression of cyclin-dependent kinase inhibitor *P21* was confirmed by quantitative reverse transcription PCR (RT-qPCR) (Figure 1b). Lamin B1 (LMNB1), a nuclear envelope marker, was also found reduced in P12 [14,15] (Figure 1b,c). Additionally, we tested the proliferation ability of replicative senescent (P12) hDPCs. Colony formation experiments confirmed that the self-renewal ability of replicative senescent (P12) hDPCs decreases (Figure 1d). To assess the differentiation potential of P12 hDPCs, we conducted osteogenic induction experiments. Culturing P5 and P12 hDPCs in osteogenic induction medium for 3 days, we observed a decrease in alkaline phosphatase (ALP) staining of replicative senescent (P12) hDPCs (Figure 1e). Consistently, we also found that expressions of osteogenic marker genes *ALP*, *RUNX2*, and *COL1A1* were downregulated in P12 hDPCs after 3 days of osteogenic induction (Figure 1f). In conclusion, hDPCs undergo replicative senescence after passage, and their proliferation and differentiation abilities decrease.

### 3.2. Serine Metabolism Decreases during Replicative Senescence in Human Dental Pulp Cells

**Figure 2:**
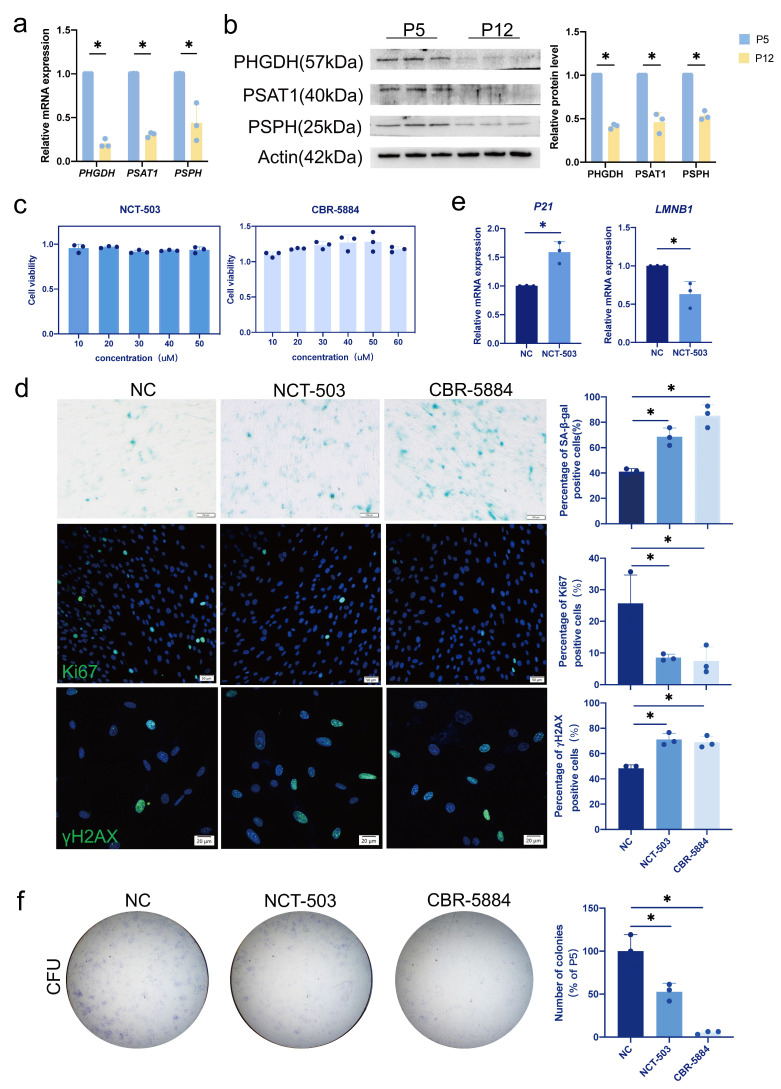
PHGDH is involved in the cellular senescence of hDPCs. (**a**) Analysis of the transcription of PHGDH, PSAT1, and PSPH in different passages (P5 and P12) of hDPCs by RT-qPCR. (**b**) Analysis of PHGDH, PSAT1, and PSPH expression in different passages (P5 and P12) of hDPCs by western blot. (**c**) Young hDPCs (P5) were treated with different concentrations of NCT—503 and CBR—5884 for 48 h, and cell viability was determined by CCK8 assay. (**d**) Effects of NCT—503 (20 μM) and CBR—5884 (30 μM) treatment for 48 h on young hDPCs (P5) were determined by SA-β-gal staining, Ki67 staining, and yH2AX staining. In SA-β-gal staining, blue-stained cells are positive cells. In Ki67 staining and yH2AX staining, the blue color in the nucleus shows DAPI staining, the green color in the nucleus shows Ki67 or yH2AX staining. (**e**) Transcription of *LMNB1* and *P21* in hDPCs (P5) treated with NCT—503 (20 μM) and CBR—5884 (30 μM) for 48 h was analyzed by RT-qPCR. (**f**) Crystal violet staining determined colony formation at 7 days after continuous treatment with NCT—503 (20 μM) and 2 days of CBR—5884 (30 μM) treatment. Data were expressed as means ± SD, *n* = 3 independent experiments. * *p* < 0.05.

To investigate the changes in serine metabolism during replicative senescence, we performed RT-qPCR detection on P5 and P12 hDPCs. We observed that levels of phosphoglycerate dehydrogenase (PHGDH), phosphoserine aminotransferase 1 (PSAT1), and phosphoserine phosphatase (PSPH), which are involved in the serine synthesis pathway (SSP), were significantly downregulated (Figure 2a,b). Of these, PHGDH, the first rate-limiting enzyme in SSP, declined the most during replicative senescence.

### 3.3. Inhibition of PHGDH Phenocopies the Replicative Senescence

Considering the decrease of PHGDH in hDPCs replicative senescence, we examined the effect of inhibiting PHGDH on young (P5) hDPCs. We selected two commonly used PHGDH inhibitors, NCT—503 and CBR—5884, and tested cell viability in P5 hDPCs. To this end, P5 hDPCs were treated with NCT-503 and CBR-5884 at different concentrations for 48 h (Figure 2c). To limit the adverse effect on cell proliferation, we used 20 μM NCT—503 and 30 μM CBR—5884 for subsequent experiments. We observed an increased percentage of senescent cells in PHGDH-inhibited hDPCs by increased SA-β-gal staining, yH2AX staining, and decreased Ki67 staining (Figure 2d). Moreover, RT-qPCR results showed that the expression of *P21* was upregulated and the expression of *LMNB1* was downregulated in PHGDH-inhibited hDPCs (Figure 2e). Colony formation experiments were also performed to evaluate the inhibition of PHGDH on stem cell renewal ability, which was also observed to decrease significantly (Figure 2f). Of note, these experiments were all performed with normal concentrations of serine in the culture medium. Thus, our results indicated that inhibition of PHGDH results in the same phenotype as replicative senescence even in the presence of serine, leading us to hypothesize that PHGDH may affect cellular senescence in addition to SSP.

### 3.4. Serine Supplementation Fails to Rescue Replicative Senescence

**Figure 3:**
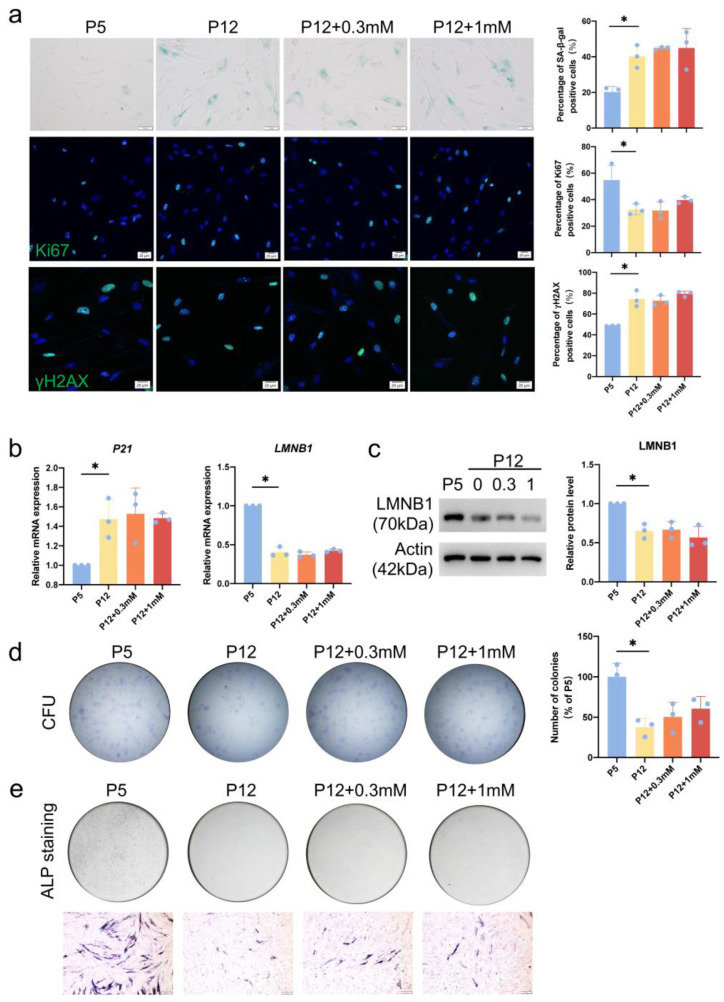
Serine supplementation fails to rescue replicative senescence. Serine was continuously supplemented during the passage process (P5 to P12 hDPCs). (**a**) Effects of adding different concentrations of serine (0.3 mM, 1 mM) to hDPCs during passage on cellular senescence were determined by SA-β-gal staining, Ki67 staining, and yH2AX staining. In SA-β-gal staining, blue-stained cells are positive cells. In Ki67 staining and yH2AX staining, the blue color in the nucleus shows DAPI staining, the green color in the nucleus shows Ki67 or yH2AX staining. (**b**) Transcription of *LMNB1* and *P21* in hDPCs supplemented with different concentrations of serine (0.3 mM, 1 mM) during passage was analyzed by RT-qPCR. (**c**) Analysis of LMNB1 expression in hDPCs supplemented with different concentrations of serine (0.3 mM, 1 mM) during passage by western blot. (**d**) Crystal violet staining determined the colony formation of hDPCs supplemented with different concentrations of serine (0.3 mM, 1 mM) during passage at 7 days. (**e**) ALP staining of hDPCs supplemented with different concentrations of serine (0.3 mM, 1 mM) during passage after 2 days of osteogenic induction. Dark bluish-purple precipitation represents Alkaline Phosphatase staining (scale bar = 200 μm). Data were expressed as means ± SD, *n* = 3 independent experiments. * *p* < 0.05.

To explore whether serine supplementation counteracted the replicative senescence of hDPCs, we continued to add serine to the culture medium during the passage process from P5 to P12 hDPCs. We observed that the percentage of senescent cells remained increasing during passaging to P12. Supplementation of neither 0.3 mM nor 1 mM serine decreased this increased percentage of senescent cells in P12 (Figure 3a). Our results confirmed that supplementation of serine did not rescue the expression of *P21* and LMNB1 in P12 hDPCs, either (Figure 3b,c). Consistently, colony formation experiments also showed that supplementation of serine did not increase the cell renewal ability in hDPCs (Figure 3d). To assess the effect of serine supplementation on the differentiation potential of replicative senescent (P12) hDPCs, we conducted osteogenic induction experiments, from which we could see that supplementation of 0.3 mM and 1 mM serine failed to rescue the differentiation potential of P12 hDPCs (Figure 3e).

### 3.5. Serine Supplementation Does Not Rescue Cellular Senescence Caused by PHGDH Inhibition

**Figure 4:**
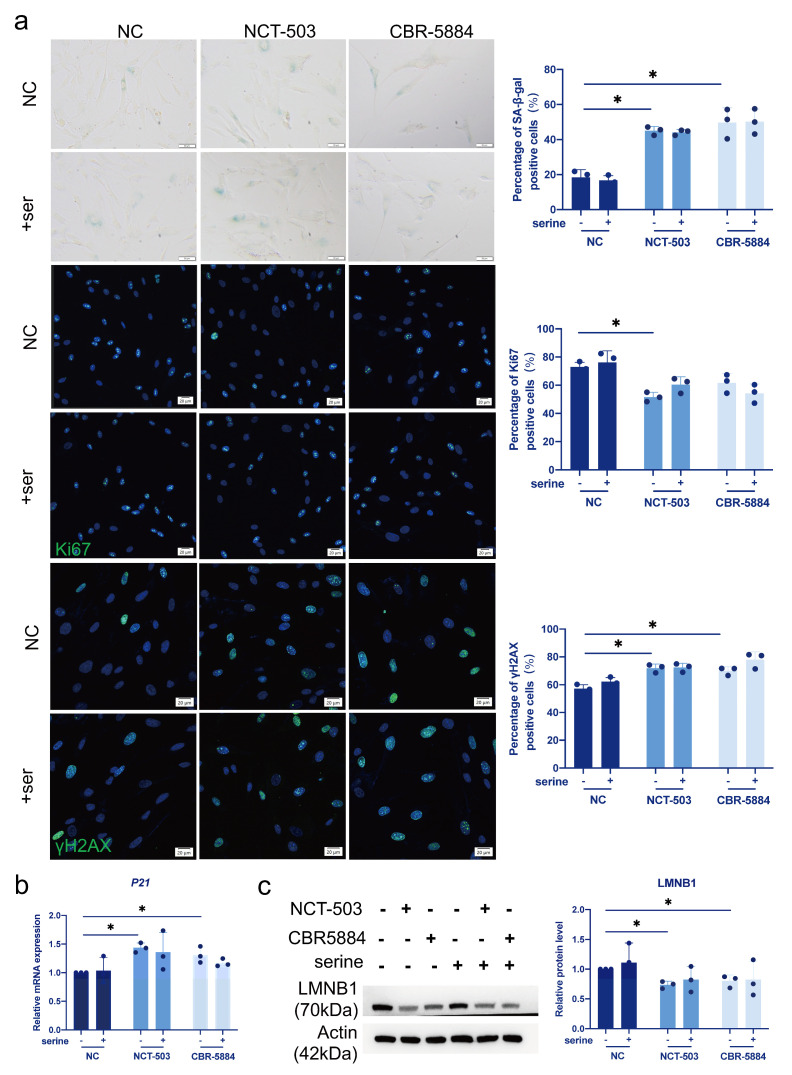
Serine supplementation does not rescue cellular senescence caused by PHGDH inhibition. Inhibitor treatment was performed in a serine/glycine-free medium. (**a**) Effects of NCT—503 (20 μM), CBR—5884 (30 μM) and serine (300 μM) treatment for 48 h on young hDPCs (P5) were determined by SA-β-gal staining, Ki67 staining and yH2AX staining. In SA-β-gal staining, blue-stained cells are positive cells. In Ki67 staining and yH2AX staining, the blue color in the nucleus shows DAPI staining, the green color in the nucleus shows Ki67 or yH2AX staining. (**b**) Transcription of *P21* in young hDPCs (P5) treated with NCT—503 (20 μM), CBR—5884 (30 μM), and serine (300 μM) for 48 h was analyzed by RT-qPCR. (**c**) Analysis of LMNB1 expression in young hDPCs (P5) treated with NCT—503 (20 μM), CBR—5884 (30 μM), and serine (300 μM) for 48 h by western blot. Data were expressed as means ± SD, *n* = 3 independent experiments. * *p* < 0.05.

To study the effect of serine on inhibitor-induced hDPCs senescence, we cultured cells in serine/glycine-free medium and treated them with inhibitors and serine. As we could observe, in a serine/glycine-free medium, inhibitor treatment caused an increased percentage of senescent cells, which could not be rescued by serine supplementation (Figure 4a). Consistently, we found that supplementing serine could not alter the expression of *P21* and LMNB1 in PHGDH-inhibited hDPCs (Figure 4b,c).

### 3.6. S-Adenosylmethionine (SAM) Regulates the Recruitment of H3K36me3 in the SIRT1 and RUNX2 Promoter Regions in Senescent Cells

**Figure 5:**
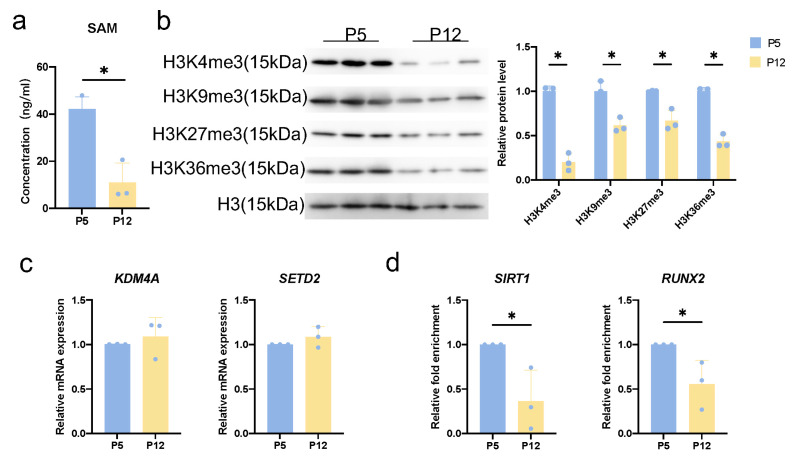
S-adenosylmethionine (SAM) regulates the recruitment of H3K36me3 in the Sirtuin 1 (*SIRT1*) and Runt-related transcription factor 2 (*RUNX2*) promoter regions in senescent cells. (**a**) Analysis of intracellular SAM concentration in young (P5) and replicative senescent (P12) hDPCs by Elisa. (**b**) Analysis of H3K4me3, H3K9me3, H3K27me3, and H3K36me3 expression in young (P5) and replicative senescent (P12) hDPCs by western blot. (**c**) Transcription of *KDM4A* and *SETD2* of young (P5) and replicative senescent (P12) hDPCs by RT-qPCR. (**d**) Analysis of H3K36me3 recruitment in *SIRT1* and *RUNX2* promoters in young (P5) and replicative senescent (P12) hDPCs by ChIP. Data were expressed as means ± SD, *n* = 3 independent experiments. * *p* < 0.05.

We hypothesized that serine, as the main donor of one-carbon units, may regulate cellular senescence through SAM and histone methylation reaction driven by it. We first detected SAM levels in hDPCs during replicative senescence and found that SAM levels significantly decreased in P12 hDPCs (Figure 5a). Furthermore, we observed that the levels of several common histone methylation modifications (H3K4me3, H3K9me3, H3K27me3, H3K36me3) were reduced (Figure 5b). Among these, we selected H3K36me3 for further investigation because there are relatively few studies on H3K36me3 regulation of cellular senescence. The only trimethyltransferase of H3K36me3, SET Domain Containing 2 (*SETD2*), and the main demethylase, Lysine-specific demethylase 4 A (*KDM4A*), showed no significant changes during replicative senescence, suggesting that the decrease in H3K36me3 was due to decreased SAM levels (Figure 5c). We then conducted ChIP experiments. We observed reduced recruitment of H3K36me3 in the promoter regions of the senescence-related regulatory gene Sirtuin 1 (*SIRT1*) and the osteogenesis-related gene Runt-related transcription factor 2 (*RUNX2*) in replicative senescent hDPCs (Figure 5d). These results indicated that SSP affects cellular senescence by regulating the recruitment of H3K36me3 in the *SIRT1* and *RUNX2* promoter regions through SAM.

### 3.7. Folate Metabolism Is Altered during Cellular Senescence

**Figure 6:**
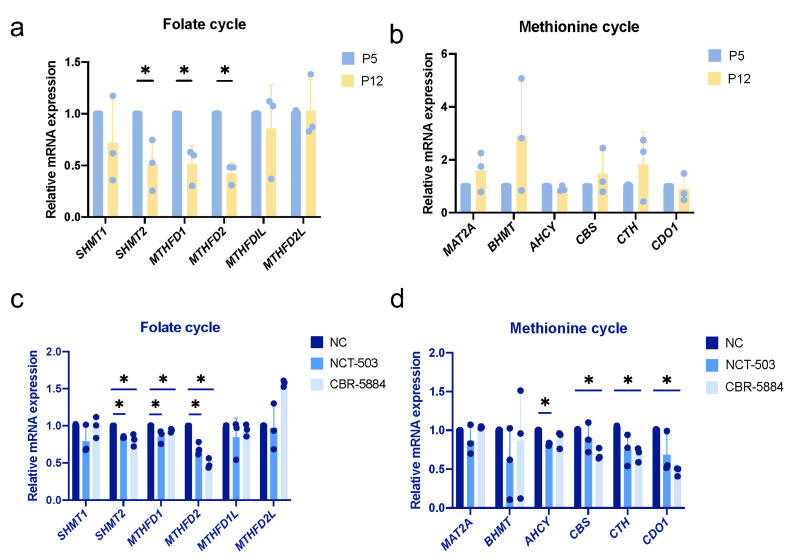
Folate metabolism is altered during cellular senescence. (**a**) Analysis of the transcription of folate metabolism-related genes (*SHMT1, SHMT2, MTHFD1, MTHFD2, MTHFD1L, MTHFD2L*) in young (P5) and replicative senescent (P12) hDPCs by RT-qPCR. (**b**) Analysis of the transcription of methionine metabolism-related genes (*MAT2A, BHMT, AHCY, CBS, CTH, CDO1*) in young (P5) and replicative senescent (P12) hDPCs by RT-qPCR. (**c**) Analysis of the transcription of folate metabolism-related genes (*SHMT1, SHMT2, MTHFD1, MTHFD2, MTHFD1L, MTHFD2L*) in young (P5) hDPCs treated with NCT-503 (20 μM), CBR-5884 (30 μM) for 48 h in serine/glycine-free medium by RT-qPCR. (**d**) Analysis of the transcription of methionine metabolism-related genes (*MAT2A, BHMT, AHCY, CBS, CTH, CDO1*) in young (P5) hDPCs treated with NCT-503 (20 μM), CBR-5884 (30 μM) for 48 h in serine/glycine-free medium by RT-qPCR. Data were expressed as means ± SD, *n* = 3 independent experiments. * *p* < 0.05.

We hypothesized that PHGDH inhibition would affect one-carbon metabolism and serine utilization, leading to a reduction in SAM production, thereby regulating gene expression through histone methylation modifications. We then detected genes related to folate and methionine cycle during replicative senescence in hDPCs and observed that serine hydroxymethyl transferase 2 (*SHMT2*), methylenetetrahydrofolate dehydrogenase 1(*MTHFD1*), methylenetetrahydrofolate dehydrogenase 2(*MTHFD2*) were significantly decreased (Figure 6a,b). Consistently, we also observed a decrease in *SHMT2*, *MTHFD1*, and *MTHFD2* in inhibitor experiments (Figure 6c,d).

## 4. Discussion

Although there have been studies on the relationship between the cellular senescence of hDPCs and serine metabolism, we are the first to introduce the concept of tissue engineering and replicative senescence. As potential seed cells, dental pulp stem cells have been used in tissue engineering and can form the dentine-pulp complex [16], repair bone defects [17,18], and promote periodontal regeneration in infrabony defects [19]. However, it requires a large amount of expansion during the application process, which causes the cells to undergo senescence and puts them at risk of developing cancer [20]. Our current results provide evidence that other metabolites derived from the de novo serine synthesis pathway may be involved, rather than serine, in the replicative senescence of hDPCs. In future studies, we will continue to screen for derivatives of serine synthesis pathway that can rescue replicative senescence to delay the senescence of hDPCs during expansion and to improve their efficacy and safety in clinical applications of tissue engineering.

Aging affects the self-renewal and differentiation potential of mesenchymal stem cells [5]. Therefore, exploring the possible mechanisms of Human dental pulp cells (hDPCs) senescence will help improve the efficacy of regenerative therapy. Here, we observe that hDPCs undergo replicative senescence after passage, and their proliferation and osteogenic differentiation abilities decrease. In this study, we only conducted early induction of osteogenesis for 3 days. In the future, we will further examine the effects of replicative senescence and inhibitor-induced senescence on the whole osteogenic differentiation process. The detection of replicative senescence in this study mostly focused on cellular senescence. In future studies, we will further use telomere-shortening experiments to verify replicative senescence.

Exogenous serine and PHGDH-mediated de novo serine synthesis are co-regulators of tumor growth and metastasis, immune cell function, etc. [21,22]. We find that PHGDH, the key enzyme of the serine synthesis pathway (SSP), is significantly decreased in replicative senescent hDPCs. However, we notice that inhibition of PHGDH results in the same phenotype as replicative senescence even in the presence of exogenous serine. Serine/glycine starvation alone does not cause cellular senescence, as it has been shown that serine starvation leads to ATF-4-dependent induction of SSP enzyme expression, allowing cells to adapt to reduced exogenous serine levels [23,24]. In addition, our study shows that the continuous addition of serine to the culture medium during the passage from P5 to P12 cannot delay the replicative senescence of hDPCs. In a serine/glycine-free medium, the inhibitor-induced senescence phenotype is also not rescued by serine. This is consistent with the research by Vandekeere et al., which shows that serine supplementation cannot rescue the proliferation defect of PHGDH knockdown cells [25]. Contrarily, another study reports that serine can partially rescue endothelial cell senescence caused by PHGDH knockdown [13]. All these indicate that PHGDH has biological functions other than serine biosynthesis.

Histone methylation plays an important role in aging and lifespan determination [26,27]. It has been reported that loss of H3K36me3 in Saccharomyces cerevisiae is associated with shortened lifespan, and the loss of H3K36me3 demethylase can extend the lifespan of Saccharomyces cerevisiae [28]. Our study finds that in replicative senescent hDPCs, the levels of the methylation donor S-adenosylmethionine (SAM), a downstream metabolite of serine metabolism, were decreased. Furthermore, H3K36me3 modification also decreases, but there are no significant changes in H3K36me3 methylase and demethylase. SIRT1, a NAD+-dependent deacetylase, is an important target in the regulation of aging, and its overexpression has been shown to reduce aging and extend lifespan in several species [29]. RUNX2 is an important determinant of osteogenic differentiation [30]. Our results indicate that H3K36me3 is involved in the senescence of hDPCs by regulating the expression of *SIRT1* and *RUNX2*.

One-carbon metabolism, including the folate and methionine cycles, is a complex metabolic network involving the biosynthesis of nucleotides and proteins to support self-renewal and proliferation [31]. Moreover, one-carbon metabolism provides methylation donors for post-translational modifications [32]. Serine is the major donor of one-carbon units [33]. PHGDH has been reported to coordinate the availability of one-carbon units of endogenous and exogenous serine in addition to its serine biosynthetic function [34]. Our study shows that in replicative senescence and inhibitor-induced senescence of hDPCs, folate metabolism-related genes *SHMT2*, *MTHFD1*, and *MTHFD2* decrease, indicating that the reduced function of PHGDH in senescent hDPCs may limit folate metabolism. This speculation requires further research to prove.

In summary, we find that PHGDH-mediated serine biosynthesis reduces H3K36me3 levels by providing less methylation donor SAM, thereby inhibiting the expression of *SIRT1* and *RUNX2* and causing hDPCs senescence. Our study demonstrates the importance of PHGDH and serine metabolism in hDPCs senescence and provides a potential target to delay cell senescence.

## Figures and Tables

**Table 1:** List of primers used in the study.

Gene	Forward Sequence	Reverse Sequence
qPCR		
*P21*	5′-GATGAGTTGGGAGGAGGCAG-3′	5′-CTGAGAGTCTCCAGGTCCAC-3′
*LMNB1*	5′-AAGCATGAAACGCGCTTGG-3′	5′-AGTTTGGCATGGTAAGTCTGC-3′
*ALP*	5′-GACCTCCTCGGAAGACACTC-3′	5′-TGAAGGGCTTCTTGTCTGTG-3′
*RUNX2*	5′-GACTGTGGTTACCGTCATGGC-3′	5′-ACTTGGTTTTTCATAACAGCGGA-3′
*COL1A1*	5′-TCTAGACATGTTCAGCTTTGTGGAC-3′	5′-TCTGTACGCAGGTGATTGGTG-3′
*PHGDH*	5′-GCAAATCTGCGGAAAGTGCT-3′	5′-ATAAGGCCTTCACAGTCCTGC-3′
*PSAT1*	5′- AAAAACAATGGAGGTGCCGC-3′	5′-GGCTCCACTGGACAAACGTA-3′
*PSPH*	5′-GTAGGGCTCTGGATGCTGC-3′	5′-TGCAAGTGCTTCTGTAAACTTAAAA-3′
*SETD2*	5′-TGCTTCTAGTCGATTTTTGCCC-3′	5′-AGGGTTTGGAGTATCACTTTGC-3′
*KDM4A*	5′-CCTCACTGCGCTGTCTGTAT-3′	5′-CCAGTCGAAGTGAAGCACAT-3′
*SHMT1*	5′-TACCCGGGCCAGAGATACTA-3′	5′-CTGAGTAGGGCTGGACGTTG-3′
*SHMT2*	5′-TTCTCTTTGTTTTGGGCGGC-3′	5′-TGTTTGCTTCCCCAGTCTGA-3′
*MTHFD1*	5′-TAGGAACGATGAGCACAATGC-3′	5′-AGACACTGGCCAGACTTTCAA-3′
*MTHFD2*	5′-TGGCTGCGACTTCTCTAATG-3′	5′-CCTTCCAGAAATGACAACAGC-3′
*MTHFD1L*	5′-CCCTTTGGTCGGAACGATGA-3′	5′-TGCCGAACACCATACTCCAC-3′
*MTHFD2L*	5′-CCAGGAGGTGATGCAACTGT-3′	5′-TCCTGTCACTGGATCGTGGA-3′
*AHCY*	5′-ATTCCGGTGTATGCCTGGAAG-3′	5′-GAGATGCCTCGGATGCCTG-3′
*BHMT*	5′-TGGAGAACAGGGGCAACTATG-3′	5′-CTGACTCACTCCTCCTGCTAC-3′
*MAT2A*	5′-GACATTGGTGCTGGAGACCA-3′	5′-ACTCTGATGGGAAGCACAGC-3′
*CBS*	5′-GATTATCGAGCCGACATCCG-3′	5′-GTCCTCACAATCTCAGCCC-3′
*CTH*	5′-TTAGCCTATTGCGTCATTTAAGCA-3′	5′-CTGCACCCGGTCATGAGTT-3′
*CDO1*	5′-TCTCTGTTGGGGTGAAGGAC-3′	5′-GCCAGGCAAATAATGTCTCC-3′
β *-actin*	5′-CCTCGCCTTTGCCGATCC-3′	5′-CGCGGCGATATCATCATCC-3′
ChIP-qPCR		
*SIRT1*	5′-AGAAACGCTGTGCTCCAGGCAGATG-3′	5′-GTAAAACGAGGGGTACCTAGTAGTTC-3′
*RUNX2*	5′-CCCAAGCTCATCTTGTACTCG-3′	5′-CCTAGAAGGGGCCTGGAA-3′

## Data Availability

Data are available from the corresponding author upon request.
